# Universal Screening for Hepatitis C Virus in the ED Using a Best Practice Advisory

**DOI:** 10.5811/westjem.2021.1.49667

**Published:** 2021-05-14

**Authors:** James S. Ford, Tasleem Chechi, Kavian Toosi, Bilawal Mahmood, Dillon Meehleis, Michella Otmar, Nam Tran, Larissa May

**Affiliations:** * UC Davis Health, Department of Emergency Medicine, Sacramento, California; † UC Davis Health, Department of Pathology and Laboratory Medicine, Sacramento, California

## Abstract

**Introduction:**

In 2019 the United States Preventive Services Task Force (USPSTF) released draft guidelines recommending universal hepatitis C virus (HCV) screening for individuals aged 18–79. We aimed to assess the efficacy of an emergency department-based HCV screening program, by comparing screening practices before and after its implementation.

**Methods:**

We performed a retrospective cohort analysis of two temporally matched, 11-month study periods, corresponding to before and after the implementation of a best practice advisory (BPA). Patients were screened for anti-HCV antibody (Ab), and positive results were followed by HCV viral load (VL) testing. The primary implementation outcome was ED testing volume (number of tests performed/month). The primary screening outcomes were the seroprevalence of anti-HCV Ab and HCV VL. We describe data with simple descriptive statistics.

**Results:**

The median age of patients was similar between periods (pre: 50 years [interquartile range [IQR] 34–62], post: 47 years [IQR 33–59]). Patients screened were more likely to be males in the pre-BPA period (Male, pre: 60%, post: 49%). During the pre-BPA study period, a total of 69,604 patients were seen in the ED, and 218 unique patients were screened for HCV (mean 19.8 tests/month). During the post-BPA study period, a total of 68,225 patients were seen in the ED, and 14,981 unique patients were screened for HCV (mean 1361.9 tests/month). Anti-HCV Ab seroprevalence was 23% (51/218) and 9% (1340/14,981) in the pre-BPA and post-BPA periods, respectively. In the pre-BPA period, six patients with a positive anti-HCV Ab level had follow-up VL testing (detectable in three). In the post-BPA period, reflex VL testing was performed in most patients (91%, 1225/1,340), and there were 563 patients with detectable VLs, indicating active infection.

**Conclusion:**

Our study shows that using a universal BPA-driven screening protocol can dramatically increase the number of patients screened for HCV and increase the number of new HCV diagnoses.

## INTRODUCTION

There are over two million individuals with chronic hepatitis C virus (HCV) in the United States (US), making it the most common bloodborne infection in the country.^1^ Due to downstream consequences of infection, such as cirrhosis and hepatocellular carcinoma, HCV is responsible for more deaths than any other chronic infectious disease in the US.^2^ With curative treatments now available, a systematic approach to identifying infected individuals could drastically reduce the burden of disease.^3^,^4^ In 2019, the US Preventative Service Task Force (USPSTF) released guidelines recommending HCV screening in all adults aged 18–79 years.^5^

The emergency department (ED) is often used by underserved, high-risk populations, making it an important setting to deliver healthcare services to patients who are not seen in traditional outpatient settings.^6^,^7^ ED-based screening programs have demonstrated success in screening for other infectious diseases such as human immunodeficiency virus (HIV) and hepatitis B virus.^6^,^8^ However, due to difficulties with patient follow-up and linkage to care, using the ED as a setting for delivery of public health interventions remains controversial. Previous studies have investigated the role of the ED in screening for HCV, in both targeted and nontargeted populations.^8^–^14^ However, few studies have explored the use of an electronic health record (EHR)-based best practice advisory (BPA) for this end.^15^

In 2018, the study institution implemented a new HCV screening protocol that used an EHR-based BPA. We aimed to assess the utility of this screening protocol, by comparing screening practices before and after its implementation.

## METHODS

### Overview

In this study, we characterize the design of the ED HCV screening program and report implementation and screening results. This study was approved under exempt status by the study site’s institutional review board Quality Improvement Self-Certification Tool.

### Study Setting and Population

The study institution was a quaternary referral, academic health system in northern California. The study ED was a Level I adult and pediatric trauma center that serves a mixed urban and rural population, and cares for more than 80,000 patients annually.

### Implementation Methods

#### Stakeholder Engagement

This program is the result of collaboration between the ED, the Division of Gastroenterology and Hepatology of the Department of Laboratory Medicine, the local county health department, and local federally qualified health centers (FQHC). The program was supported by funding from the Gilead Sciences, Inc. (Foster, CA) FOCUS program. The objective was to increase diagnosis of HCV. Pre-implementation activities included engaging key hospital stakeholders such as hospital leadership, ED and outpatient clinicians, laboratory leadership, representatives from information technology (IT), and local FQHCs. Three months prior to the implementation of the screening program, structured educational initiatives were performed for residents, faculty, nurses, and technicians during faculty and departmental meetings, as well as at pre-shift huddles.

**Population Health Research Capsule**

What do we already know about this issue?

*Hepatitis C virus (HCV) screening guidelines recommend screening adults aged 18–79 years. HCV testing has been explored in the ED but remains controversial*.

What was the research question?

*What is the utility of a universal best practice alert-based ED HCV screening program?*

What was the major finding of the study?

*A universal best practice alert-based ED HCV screening program drastically increased HCV testing and diagnosis*.

How does this improve population health?

*The ED has a high-risk population with HCV prevalence well above the national average. ED screening programs could improve diagnosis and linkage to treatment*.

### Reflex Laboratory Testing

We developed an onsite pathway to provide antibody screening with reflex testing for HCV RNA viral load (VL) among those specimens identified as being HCV Ab seropositive. A new ED HCV screen with reflex test was created in the study institution EHR, and implemented alongside the BPA. Under the new process, when an ED HCV screen with reflex test was ordered, the Ab screen was performed using a chemiluminescent immunoassay (Architect i1000, Abbott Laboratories, Abbott Park, IL) that detects antibodies in blood specimens. Results are reported in the EHR within 1–3 days. Any positive Ab screen underwent reflex diagnostic confirmation using an automated HCV RNA (Cobas VL assay AmpliPrep/TaqMan, Roche Diagnostics, Basel, Switzerland). The follow-up VL test result was routinely available within four days after a positive Ab screen.

#### ED Screening Program Design

**Figure 1:**
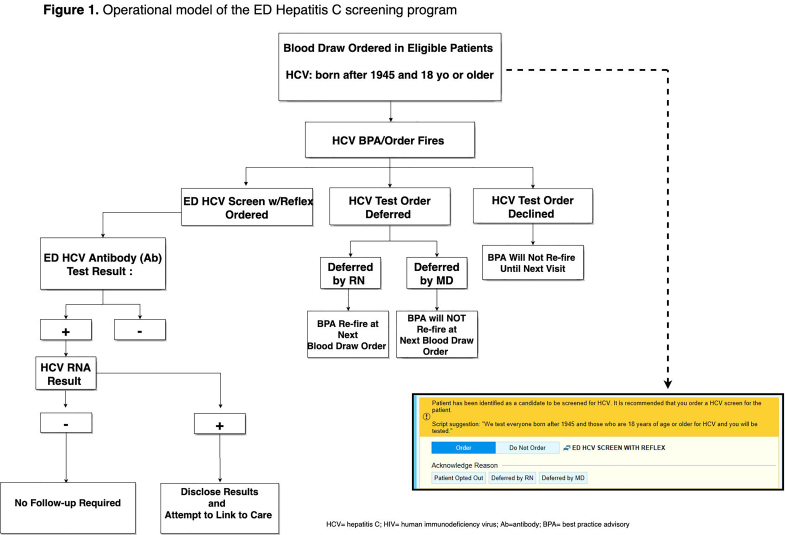
Operational model of the hepatitis C screening program in the emergency department. *HCV*, hepatitis C; *HIV*, human immunodeficiency virus; *Ab*, antibody; *BPA*, best practice advisory.

All ED patients ≥18 years and born after 1945, who were having blood drawn for any clinical purpose and who did not have a positive HCV RNA test result in the EHR, underwent opt-out HCV screening (Figure 1). Upon entering any laboratory order into the EHR, a BPA alerted the ED provider (nurse, nurse practitioner, physician, resident, fellow) that the patient was eligible for HCV screening. This BPA functioned as both an alert and a hard stop for which providers were required to respond to continue with the order entry. The ED provider could, on behalf of the patient, accept, or defer testing. If deferred by the nurse, the BPA would appear again on subsequent phlebotomy orders and if deferred by the physician, it would not appear for the duration of the current encounter but would reappear on subsequent ED visits. On the other hand, if accepted, the BPA generated HCV screening discharge documentation documenting patient verbal authorization for testing, and triggered an order in the EHR for HCV testing and printed labels for specimen collection.

To standardize screening and to comply with ethical regulations, ED providers followed a script provided on the BPA advisory. Patients were allowed to refuse testing after they were informed about the program. Information about the cost of the test was given upon patient request; otherwise, a statement about test charges was included in the patient’s discharge documents and on brochures and posters throughout the ED. Funding for the laboratory tests was obtained by charging the patient’s insurance, a billing strategy employed by similar screening programs and studies.15 There was not a way to prospectively identify which insurances would cover the test, so this information was not available to the provider or the patient to aid in the decision to offer/accept testing. If a patient requested that their insurance not be charged, or they did not have insurance, testing was paid for by the program grant. Funding for the development of the laboratory reflex pathway, IT changes to the EHR, and support for patient care navigators came from the program grant. Program staff, including two patient navigators, contacted the patients with results via telephone or in person depending on a patient’s disposition.

### Study Methods

#### Study Design

We performed a retrospective analysis of two timematched, 11-month study periods, corresponding to before and after BPA implementation. We consecutively included all patients who underwent HCV testing in the ED, in both the pre- and post-BPA study periods. The pre-BPA study period was January–November 2018. HCV screening during the pre-BPA period was clinician-initiated. The BPA was implemented on November 27, 2018, and was followed by a one-month transitional period that allowed clinicians to adjust to using the BPA, as well as to study temporally matched cohorts. The post-BPA period was January 2019–November 2019. Data were abstracted directly from the EHR using computer-generated reports. Personnel responsible for procuring these reports were blinded to the hypothesis of the study. Data elements abstracted included age, gender, race/ethnicity, chief complaint, past medical history, problem list, substance use history, insurance status, and results of HCV testing. To prevent duplicate data, only a patient’s first ED visit where they received HCV testing was included in our analysis. We stored data in de-identified datasets, and each patient was given a unique identifier to maintain patient confidentiality.

#### Implementation Outcomes

The primary outcome of the ED screening program was ED testing volume (number of tests performed/month). Secondary outcomes included the number of BPA fires and the number of BPA fires that were accepted and resulted in HCV testing.

#### Screening Outcomes

Screening outcomes included rates of positive HCV Ab and RNA results (number positive/number tested).

#### Analysis

We described data with simple descriptive statistics. Categorical variables were expressed as percentages and proportions and continuous variables were expressed as means or medians (Q1–Q3). We used Mann-Whitney U test to compare continuous variables and Fischer’s exact test to compare categorical variables. All statistical analyses were performed using Stata 15.1 (StataCorp LLC, College Station, TX).

## RESULTS

### Characteristics of Study Subjects

Patient characteristics stratified by study period are summarized in Table 1. The median age of patients was similar between periods (pre: 50 years [interquartile range (IQR) 34–62], post: 47 years [IQR 33–59]). Patients screened were more likely to be male in the pre-BPA period (male, pre: 60%, post: 49%). The proportions of patients within each racial or ethnic category were similar between study periods.

### Implementation Results

**Figure 2:**
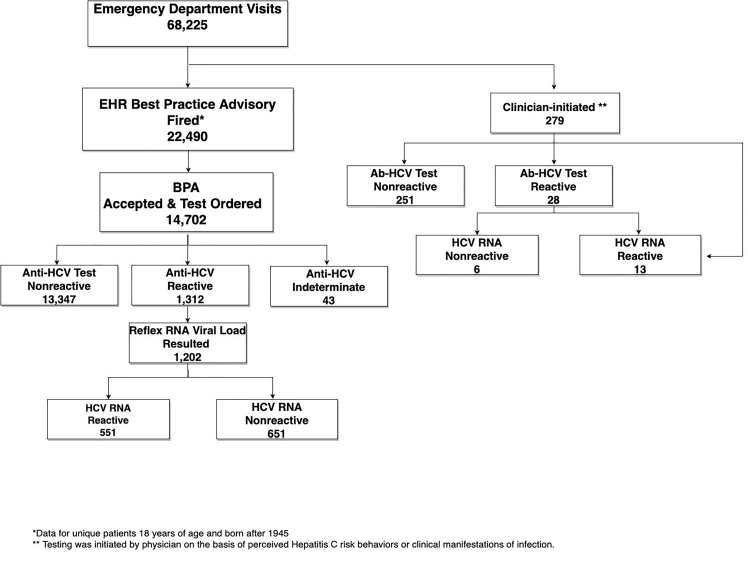
Hepatitis C virus testing schematic for the post-best practice advisory period. *Data for unique patients 18 years of age and born after 1945. **Testing was initiated by physician on the basis of perceived Hepatitis C risk behaviors or clinical manifestations of infection. *EHR*, electronic health record; *BPA*, best practice advisory; *ABHCV*, antibody-hepatitis C virus.

During the pre-BPA study period, a total of 69,604 patients were seen in the ED, and 218 unique patients were screened for HCV (mean 19.8 tests/month). During the post-BPA study period, a total of 68,225 patients were seen in the ED, and 14,981 unique patients were screened for HCV (mean 1361.9 tests/month), representing a 68-fold increase in HCV screening following BPA implementation. During the post-BPA period, the BPA was triggered in 22,490 patients and was accepted by patients and providers in 14,702 patients (65%). The BPA was deferred by providers in 61% of non-accepted BPAs (4,715/7,788) and refused by patients in 15% of nonaccepted BPAs (1,155/7,788). The reason for BPA deferment was unknown in 25% of non-accepted BPAs (1,918/7,778). Most patients in the post-BPA period were screened via the BPA (BPA-initiated: 98%, 14,702/14,981 vs. clinicianinitiated: 2%, 279/14,981). A full testing schematic for the post-BPA period is available in Figure 2.

### Screening Results

Anti-HCV seropositivity was high in both periods (pre: 23% [51/218] vs post: 9% [1340/14,981]) (Table 2). In the pre-BPA period, only 12% (6/51) of patients with a positive anti-HCV Ab level had follow-up VL testing. Three of these patients had detectable VLs, amounting to three confirmed ED diagnoses of HCV in the pre-BPA period. In the post-BPA period, reflex VL testing results were available in most patients (91%, 1225/1340). There were 563 new confirmed diagnoses of HCV during the post-BPA period, representing a 187-fold increase in diagnoses following BPA implementation. Ninety-eight percent (551/563) of HCV diagnoses in the post-BPA period were made via BPA-initiated testing, and 2% (12/563) were made via clinician-initiated testing.

Since most patients with a positive HCV Ab test in the pre-BPA period did not have follow-up VL testing, the prevalence of HCV in this cohort cannot reliably be calculated. The prevalence of HCV in the post-BPA period was 3.8% (563/14,981).

## DISCUSSION

The annual number of new cases of HCV is increasing in the United States.^16^ This increase has been driven in part by the national opioid crisis, which has led to a concomitant rise in injection drug use, resulting in more chronic HCV infections in young individuals.^17^ These individuals comprise a high-risk population, and are less likely to access primary care services and are more likely to seek care in acute care settings.^18^ As such, the ED could be an important setting to test individuals for HCV. In this study, we demonstrate that the implementation of a BPA-based screening protocol in the ED can increase HCV screening and diagnosis.

Our study population had an unexpectedly high rate of HCV RNA positivity (post-BPA: 3.8%), representing a value nearly four-fold higher than the national average (~1%).^19^ This suggests that the ED may be a high-yield setting to screen individuals for HCV. An additional 1342.1 patients/month (pre: 19.8, post: 1361.9) were screened for HCV after BPA implementation, an increase similar to the outcome in another study that explored the use of a BPA for ED-based HCV screening.^15^ Since the number of patients who underwent clinician-initiated testing did not drastically increase between study periods (pre: 218, post: 279), this suggests that the increase in testing was directly attributable to the implementation of the BPA. The BPA was accepted in 65% of patients in which it fired, a much higher rate than another BPA-based ED screening study that reported a BPA acceptance rate of 40%.^15^ This difference in BPA acceptance rate between studies may possibly be explained by differences in demographics between study institutions (ie, higher rates of government-payer insurance [72% vs 61%]), which may have influenced providers’ perceptions of a patient’s likelihood to follow up, and therefore likelihood to accept the BPA.

Unfortunately, as a VL reflex order was not in place during the pre-BPA period, the prevalence of HCV could not be calculated for this cohort. However, the seroprevalence of anti-HCV antibody was 23%, over two-fold higher than the post-BPA group (9%), and over three-fold higher than the aggregated prevalence of 19 ED-based studies (7.5%).^20^ This demonstrates that the study institution services an exceptionally high-risk population. It also suggests, unsurprisingly, that universal screening may be less discriminate in screening for HCV, compared to providers who test patients based on their clinical presentation and risk factors for infection.

While universal screening may be less selective than clinician judgment, the increased testing following BPA implementation led to the identification of 550 more cases of HCV in a temporally matched 11-month period. This is commensurate with previous studies that demonstrate that technology-based infectious disease screening strategies are more effective than provider-driven protocols, in an ED setting.^10^ With HCV testing becoming more affordable and curative therapy now available, the benefit of early detection with linkage-to-treatment in individuals with chronic infection likely outweighs the cost of increased testing.^21^

To assess the impact of provider bias on screening practices we compared demographic proportions of screened individuals before and after the implementation of the BPA. In the pre-BPA period, individuals tested were more likely to be male (60%). However, after implementation of the BPA, patient gender was evenly distributed between males and females (males: 49%). This suggests that in the absence of a BPA-driven screening protocol, females may not be offered HCV screening as often as males.

## LIMITATIONS

Our study must be interpreted in light of its limitations. This study was retrospective; thus, we were limited by the data in the EHR. This was a single-institution study at a large, academic center with a mixed urban and rural population; hence, our findings may not be generalizable to all settings. The BPA was introduced alongside a new EHR order that automatically ordered VL reflex testing for reactive HCV Ab testing; so it is difficult to separate the effect of the BPA from the new reflex order. Linkageto-care data was not available at the time of this study, so we cannot evaluate the full impact of screening in this study. Future studies will coordinate with primary care and hepatology clinics to obtain linkage-to-care data. Additionally, future studies will examine clinician perceptions related to BPA-implementation.

## CONCLUSION

Our study shows that using a universal BPA-driven screening protocol can dramatically increase the number of patients screened for HCV and increase the number of new HCV diagnoses. We also demonstrate that a BPA-driven screening protocol may help reduce provider and genderbased biases, and increase screening in females. Using this ED-based approach for HCV screening could help combat the rise in HCV, particularly in individuals without access to other forms of healthcare.

## Figures and Tables

**Table 1:** Patient characteristics by study period.

Characteristic	Pre-BPA (n = 218)	Post-BPA (n = 14,981)	P-value
Age (years)	50 (34–62)	47 (33–59)	0.09
Gender^1^			
Male	130 (60%)	7,273 (49%)	<0.001
Female	88 (40%)	7,706 (51%)	
Race/ethnicity^2^			
White	135 (64%)	8,970 (60%)	0.40
Black	38 (18%)	2,903 (20%)	0.60
Asian	9 (4%)	1,124 (8%)	0.07
Mixed/other	30 (14%)	1,784 (12%)	0.34
Hispanic	34 (17%)	3,351 (23%)	0.05

Age reported as median (Q1–Q3) and analyzed between study periods using Mann-Whitney U test. Categorical variables reported as number (%) and analyzed between study periods using Fisher’s exact test.

1 Gender data missing for two patients in post-BPA period.

2 Race data missing for 6 patients in pre-BPA and 200 patients in post-BPA group. Ethnicity data (Hispanic vs non-Hispanic) were missing in 16 patients in the pre-BPA group and 177 patients in the post-BPA group.

*BPA*, best practice advisory.

**Table 2:** Hepatitis C virus test results by screening period.

	Pre-BPA	Post-BPA	P-value
Anti-HCV Ab	N = 218	N = 14,981^2^	
Reactive	51 (23%)	1,340 (9%)	<0.001
Nonreactive	166 (76%)	13,598 (91%)	
Indeterminate	1 (<1%)	43 (<1%)	
HCV VL^1^	N = 6	N = 1,225	
Detected	3 (50%)	563 (46%)	1.0
Not detected	3 (50%)	662 (54%)	

Values expressed as percentage (number). Comparisons between study periods made via Fisher’s exact test.

1 Reflex viral load testing was not performed during the pre-BPA period. Reflex VL testing was not available for 106 patients in the post-BPA group who underwent anti-HCV Ab testing.

2 Includes 14,702 patients tested via BPA-initiated testing and 279 patients tested via clinician-initiated testing.

*Ab*, antibody; *BPA*, best practice advisory; *HCV*, hepatitis C virus; *VL*, viral load.
